# Perspective Distortion from Interpersonal Distance Is an Implicit Visual Cue for Social Judgments of Faces

**DOI:** 10.1371/journal.pone.0045301

**Published:** 2012-09-21

**Authors:** Ronnie Bryan, Pietro Perona, Ralph Adolphs

**Affiliations:** 1 Computation and Neural Systems Program, California Institute of Technology, Pasadena, California, United States of America; 2 Division of Biology, California Institute of Technology, Pasadena, California, United States of America; Ecole Normale Supérieure, France

## Abstract

The basis on which people make social judgments from the image of a face remains an important open problem in fields ranging from psychology to neuroscience and economics. Multiple cues from facial appearance influence the judgments that viewers make. Here we investigate the contribution of a novel cue: the change in appearance due to the perspective distortion that results from viewing distance. We found that photographs of faces taken from within personal space elicit lower investments in an economic trust game, and lower ratings of social traits (such as trustworthiness, competence, and attractiveness), compared to photographs taken from a greater distance. The effect was replicated across multiple studies that controlled for facial image size, facial expression and lighting, and was not explained by face width-to-height ratio, explicit knowledge of the camera distance, or whether the faces are perceived as typical. These results demonstrate a novel facial cue influencing a range of social judgments as a function of interpersonal distance, an effect that may be processed implicitly.

## Introduction

We glean a wealth of socially relevant information from faces in the blink of an eye: gender, emotion, and whether a person is attractive, competent, threatening, or trustworthy, to mention a few. For example, reliable judgments of trustworthiness can be made from faces viewed for 100 ms or less [1], and such judgments are found to influence real world behavior, such as voting [2], interest rates on person to person loans [3], and behavior in economic trust games [4]. Multiple factors influence such judgments. The perceived valence of an otherwise neutral face, for example, is thought to influence trait attributions by activating brain systems tuned to facial expression [5]. The width-to-height ratio of a face has been shown to be a reliable indicator of testosterone level and linked to untrustworthy behavior [6]. Similarly, features such as the roundness of the cheeks and the large eye size (“babyfacedness”) may influence perceived trustworthiness by activating representations related to the perception of age [7]. These avenues of investigation all attempt to explain why some individuals are perceived as more or less trustworthy than others on first glance. Yet there is one important ecological cue that, to our knowledge, has not been investigated: the perspective distortion as a function of viewing distance. The change in appearance of an individual due to interpersonal distance is independent of other factors such as facial expression, subtended visual angle, or overt knowledge of interpersonal distance, and we find that it is sufficient in itself for influencing several social judgments including perceived trustworthiness. Our approach expands the investigation from an analysis of the appearance of a face to an analysis of the relationship between a viewer and another person.

**Figure 1:**
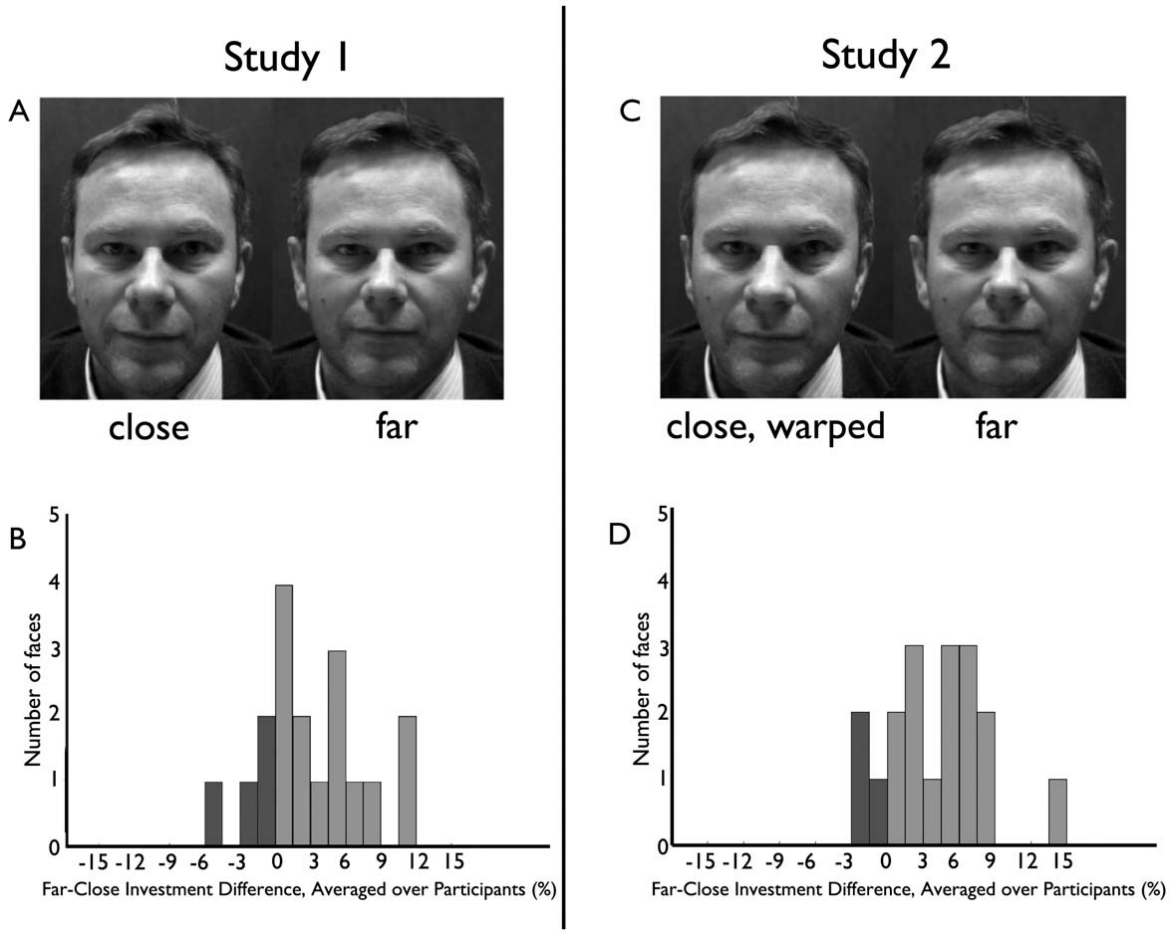
Perspective distortion from distance influences trust (Experiment 1a and Experiment 2). Histograms show investment difference (far-close) for each face, averaged over all participants. A disproportionately larger number of faces received a positive investment difference (light bars) compared to those receiving a negative investment difference (dark bars).

Three-dimensional objects, such as the human face, produce on the retina a two-dimensional image via perspective projection. The image varies with distance from the center of projection, even when equated for size (see Figure 1a); e.g., the nose looks relatively larger and the ears smaller as the distance decreases [8]. Such differences may be modeled as a distance-dependent image warp or distortion (see Figure 1c). This effect may have been utilized in portrait paintings not only to induce distance percepts but also to manipulate how viewers feel about the face [9].

Ever since Edward Hall's seminal book on the topic [10], interpersonal distance and personal space have been highlighted as ubiquitous and potent determinants of a wide variety of social behaviors [11]. Notably, interpersonal distance is associated with arousal [12], self-protective behavior [13], privacy [14], emotional valence [15], [16], management of stress and aggression [17], and interpersonal trust [18]. In each of these studies, interpersonal distance is manipulated in an ecologically valid way, that is, participants are observed reacting to a confederate standing at an experimentally determined distance. The result is that the observed changes may result from any or all of the many multi-modal perceptions that accompany a change in interpersonal distance. For example, the size of the face is smaller and the visibility of the body is greater at greater distances. These studies demonstrate the efficacy of interpersonal distance at eliciting a variety of emotional responses relevant to social judgments, but they do not yet isolate the specific perceptual cues that are responsible.

Judging socially relevant traits from faces can occur automatically and can elicit reliable ratings even after a very brief exposure [19], [1], suggesting there are processes specialized for rapid social evaluation. Interpersonal distance is a potent variable influencing social behavior [10], [11], [20], and is related to activity in several brain structures notably including the amygdala: damage to the amygdala can abolish normal interpersonal distancing behaviorally, and even the knowledge of interpersonal closeness is sufficient to drive activation of this brain structure [21]. The amygdala has also long been implicated in the automatic evaluation of threat [22], [23], facial valence information [24], and trustworthiness of faces [25], [26], [27]. Given all the varied studies briefly reviewed above, we hypothesized that the distance-dependent perspective projection of a face would be a cue for social judgments, especially those related to trust.

Since interpersonal distance is likely to influence a variety of trait judgments, we investigated a broad set of questions in these experiments, as well as having a primary focus on trustworthiness. Participants not only performed a trust game, but also rated faces on dimensions of apparent trustworthiness, competence, attractiveness, age, weight, and averageness. These attributes were selected because they have been shown to be reliable social judgments made from faces, or might be expected to vary with distance in some way.

We investigated the effects of perspective projection in three experiments that obtained social judgments (ratings) as well as measured trust behavior in terms of the amount of money participants were willing to invest in a person whose face they saw (see Table 1 for summary of experiments). The first experiment used photographs taken from different distances, while controlling the size and facial expression of the stimuli; the second used synthetically warped face images to eliminate possible confounds in highlights and focus; the third explored a number of follow-up questions with a larger subject sample tested over the internet; and the fourth replicated our effect with an entirely new set of face stimuli to establish its reliability. All effects are reported as the within-subject difference of the behavioral response to far as compared to close face stimuli (normalized within each subject). Although participant gender was not a factor of interest in our study, all findings were followed up with exploratory ANOVAs that included participant gender as a possible factor. Means and the full width of the 95% confidence interval are reported, together with Cohen's d for effect sizes.

**Table 1:** Summary of experiments.

Experiment	N	Age	Task
Experiment 1a	23	33.26±2.92	In lab: Economic Trust Game
Experiment 1b	45	29.91±1.18	In lab: Ratings: Trust, Attractiveness, Competence
Experiment 1c	37	26.38±1.45	In lab: Ratings: Heaviness, Age, Distance
Experiment 2	27	23.93±1.09	In lab: Economic Trust Game (warped faces)
Experiment 3a	268	31.5±0.62	Online: Experiment 1b Ratings
Experiment 3b	70	30.32±1.3	Online: Experiment 3a Ratings with Verbal Cue
Experiment 3c	60	32.15±1.48	Online: Experiment 3a Ratings with Size Cue
Experiment 3d	253	31.83±0.64	Online: Experiment 1c Ratings
Experiment 3e	134	31.46±0.88	Online: Averageness Ratings
Experiment 4	31	31.79±1.55	In lab: Ratings: Trust, Attractiveness, Competence

The table breaks down each experiment in terms of the number of participants (N), their age (mean and SEM) and the task used.

## Experiment 1

### Results

In Experiment 1a, faces photographed at the far distance elicited higher monetary investments in an economic trust game than those photographed at the close distance (Fig. 1a): mean investment difference (far faces - close faces) was 3.2±2.45 (95% CI), t(17) = 2.78, p<0.02 (paired t-test, 2-tailed), with an effect size of Cohen's d = 0.28. Similarly, in Experiment 1b the far faces elicited higher ratings of attractiveness (5.25±2.66 (95% CI), t(17) = 4.16, p<0.001, Cohen's d = 0.31), competence (2.49±2.48 t(17) = 2.12, p<0.05, Cohen's d = 0.20), and trustworthiness (2.82±2.67, t(17) = 2.23, p<0.05, Cohen's d = 0.24), compared with those photographed at the closer distance.

We examined the stimulus-by-stimulus correlations between the trait ratings from Experiment 1b among one another, and also with the investments made in Experiment 1a. In Experiment 1b, Trust ratings were strongly correlated with competence ratings (r(34) = 0.90, p<0.001) and attractiveness ratings (r(34) = 0.82, p<0.001). Competence and Attractiveness ratings were likewise correlated (r(34) = 0.74, p<0.001). These correlations are so high that the residual trust ratings after regressing out the ratings of attractiveness and competence no longer show a statistically significant difference between close and far faces. From Experiments 1a and Experiment 1b, investments in the trust game were correlated with ratings of trust (r(34) = 0.84, p<0.001), competence (r(34) = 0.86, p<0.001), and attractiveness (r(34) = 0.65, p<0.001). Again, residual investments after regressing out these independent face ratings no longer show a statistically significant difference between close and far faces.

In Experiment 1c, we obtained ratings of age, weight, and camera distance, which showed no statistically significant effects of age (0.14±0.64 (95% CI), t(17) = 0.22, p = 0.83. However, ratings of weight revealed that faces photographed farther away appeared heavier (3.85±2.56 (95% CI), t(17) = 3.17, p<0.01, Cohen's d = 0.19), and, paradoxically, ratings of distance revealed that the farther faces actually appeared closer (−3.06±2.65 (95% CI); t(17) = −2.43, p<0.03, Cohen's d = −0.38).

Experiment 1a investment residuals after regressing out each of these ratings from Experiment 1c do still display a statistically significant preference for faces (regressing out age: mean investment difference = 4.63±2.82 (95% CI); t(17) = 3.47, p<0.005, Cohen's d = 0.42; regressing out distance: mean investment difference = 3.97±2.36 (95% CI); t(17) = 3.55, p<0.005, Cohen's d = 0.35; regressing out weight: mean investment difference = 3.23±2.44 (95% CI); t(17) = 2.79, p<0.02, Cohen's d = 0.28).

Post-experiment debriefing confirmed that none of the participants noticed that face distance was manipulated. Finally, to explore possible gender effects, a 2×2 (participant gender x viewing distance) ANOVA on the trustworthiness ratings confirmed a significant effect of viewing distance (F(1) = 6.68, p<0.02), but failed to find a main effect or interaction of gender (F(1)<0.3, n.s.).

### Discussion

Faces photographed from within personal space elicited lower monetary investments and lower ratings of trustworthiness, attractiveness and competence than did simultaneously photographed faces from outside of personal space. All three ratings were highly correlated, as is typical, suggesting that the influence of personal space on social judgments may have a wide-ranging influence on social judgments.

The finding that the faces appear heavier is consistent with the vertically oblong shape of the human head, which will produce the greatest perspective distortion at the sides. The effect is that the width-to-height ratio is smaller for closer faces, making them appear thinner. The fact that participants rated far faces as heavier confirms they were able to physically distinguish the far faces from the close faces, but when asked explicitly about camera distance, they judged this incorrectly. The fact that viewers rated the far faces as closer suggests they may have been using a size heuristic to judge closeness, since the far faces are slightly wider than the close faces. The difference in image size obtains from the fact that distance between the eyes was used to normalize the size of the faces. We chose to normalize based on the distance between the eyes in order to prevent a change in the position of the eyes between conditions, which may have been easily noticed by the participants. The possibility that participants relied on a size cue rather than the relative facial proportions to determine distance, but are still influenced by those facial proportions when making investment and rating decisions, suggests that the evaluation of perspective projection may be processed implicitly.

## Experiment 2

It is conceivable that subtle differences in highlight and focus between the far and near pictures, independent of distance-induced warping, might contribute to this finding. More closely photographed faces exhibit a greater sheen on the highlights than do farther faces. Although the global contrast may be equalized by adjusting the dynamic range of the image, the local contrast in face areas that receive more direct illumination may still contain luminance-based cues. Similarly, closer facial features such as the nose may be photographed with a slightly different sharpness of focus than the farther features such as the ears due to the varying distance to the lens. To completely isolate perspective warp as the factor against these possible confounding variables, we repeated the economic trust game of Experiment 1a with synthetically warped faces.

### Results

The mean investment difference for synthetically warped faces (far-close faces) was 4.2±2.1 (95%CI), t(17) = 4.2, p<0.001, Cohen's d = 0.36, confirming the effect observed in Experiment 1a (Figure 1b). Post-experiment debriefing again verified that none of the participants noticed that face distance was manipulated. To explore possible gender effects, a 2×2 (participant gender x viewing distance) ANOVA showed a significant effect of viewing distance: F(1) = 15.76, p<0.001, but no effects of participant gender or interaction with gender (F<1.3; n.s.).

### Discussion

Experiment 2 confirmed that distance-induced warping alone (perspective projection) influences trust-related investment behavior even when controlling for luminance based cues such as local contrast and focus. This result does not rule out these cues as possible contributing factors, but does show that they are not necessary to obtain the effect we observed. The results of Experiment 2 demonstrate that perspective projection warping is sufficient to influence trust game behavior, opening the door for the manipulation of images even in the absence of the simultaneous photographic set-up we devised for these experiments.

## Experiment 3

Several further questions were followed up in a series of experiments administered to larger samples of participants tested over the internet. Can the effect measured in Experiments 1 and 2 be obtained with explicit distance cues, such as mere verbal information or image size? Participants seem not to be aware of any manipulation of camera distance, and so it is unclear if similar results might obtain when people are consciously aware of distance. Might the effect be due to how average (typical) the images appear? It is conceivable that we have more exposure to faces at a further distance and that this contributes to the effect we found. Is the effect sufficiently robust to appear outside the laboratory? Experiments 1 and 2 tested participants in the lab under well controlled conditions, but it would be important to establish that the effect is robust enough to influence people under less controlled circumstances that they might encounter in everyday life. We explored all these questions in Experiment 3.

Experiment 3a and Experiment 3d seek to replicate our original findings when the task is administered over the internet; Experiment 3b provides participants with explicit information about interpersonal distance from verbal information, and Experiment 3c provides visual size information.

Another potentially mediating variable that could explain the results of Experiment 1 and 2 is the typicality of the face. Averageness of faces is known to influence a host of cognitive functions [28], including the perception of attractiveness [29], so it is possible that the close faces of Experiment 1 and 2 were seen as less attractive and trustworthy simply because they were seen as less average. If participants do in fact view the faces as less average, they should be able to report this perception, as they do in other experiments [30]. Experiment 3e obtains averageness ratings to determine if this perception might account for the effect of viewing distance.

### Results

**Figure 2:**
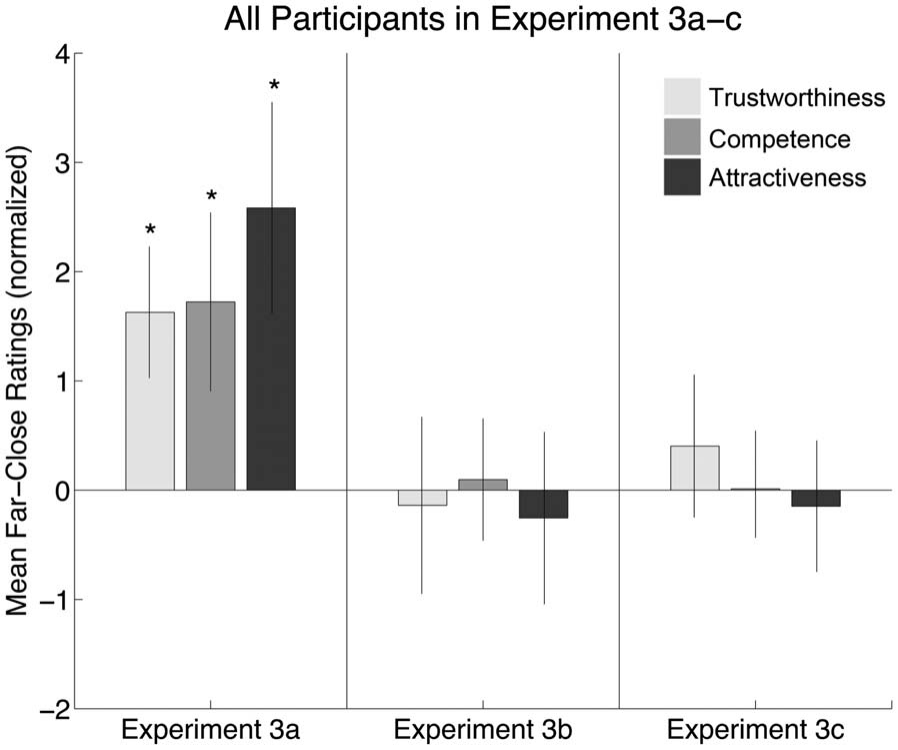
Social judgments as a function of perspective distortion (Experiment 3a), verbal information (Experiment 3b), and image size (Experiment 3c). In each Experiment, ratings were obtained for Trust (solid black bars), Competence (gray bars), and Attractiveness (white bars). The mean Far-Close score over all participants and stimulus faces is shown on the y-axis (with the error bar indicating the 95% confidence interval).

**Figure 3:**
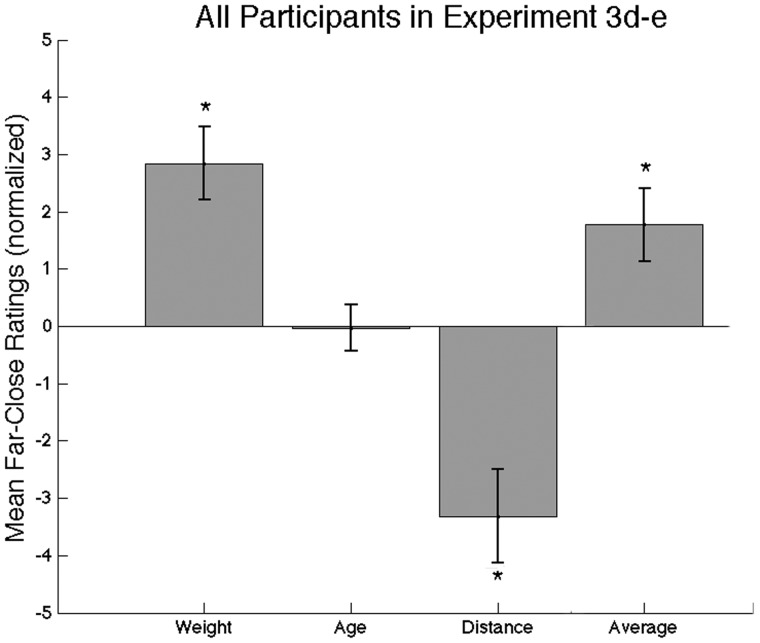
Additional social judgments from perspective distortion (Experiment 3d–e). Shown are means and 95% CI for ratings of Heaviness, Age, Distance to Camera (Experiment 3d), Averageness (Experiment 3e).

Experiment 3a replicated the effects observed in Experiment 1 for social judgments of far-close faces, trustworthiness: 1.64±1.25 (95%CI), t(17) = 2.77, p<0.02, Cohen's d = 0.15; competence: 1.76±1.68, t(17) = 2.21, p<0.05, Cohen's d = 0.14; attractiveness: 2.61±1.67, t(17) = 2.21, p<0.02, Cohen's d = 0.23. As before, 2×2 (participant gender x viewing distance) ANOVAs confirmed a significant effect of viewing distance: trustworthiness: F(1) = 14.4, p<0.001; competence: F(1) = 12.0, p<0.001; attractiveness: F(1) = 44.6, p<0.001, but no effects of participant gender or interaction with gender (all F<0.6; n.s.). See Figures 2,3 for a summary of the results of Experiment 3.

As with Experiment 1, we examined the correlation between the trait ratings and the investments. Investment amounts were highly correlated with these independent ratings of trustworthiness (r(34) = 0.86, p<0.001), competence (r(34) = 0.88, r<0.001), and attractiveness (r(34) = 0.66, p<0.001). Once again, the investment residuals after regressing out each of these ratings did not display a statistically significant preference for far faces.

Experiment 3b showed no effect of explicit verbal information about distance on any rating: competence: 0.16±1.16, t(17) = 0.30, p = 0.77, Cohen's d = 0.0084; trust: −0.14±1.65, (t(17) = −0.18, p = 0.86, Cohen's d = −0.021; attractiveness: −0.24±1.59, t(17) = −0.32, p = 0.75, Cohen's d = −0.024.

Experiment 3c showed no effect of image size: competence: −0.069±0.90, t(17) = −0.16, p = 0.84, Cohen's d = 0.00027; trust: 0.37±1.31, (t(17) = 0.60, p = 0.55, Cohen's d = 0.0029; attractiveness: −0.17±1.23, t(17) = −0.30, p = 0.77, Cohen's d = −0.016.

Experiment 3d replicated Experiment 1c findings (heaviness: 2.86±1.30 (95% CI), t(17) = 4.63, p<0.0005, Cohen's d = 0.14; distance −3.30±1.67 (95% CI), t(17) = −4.15, p<0.0001, Cohen's d = −0.87), as well as the lack of an effect for age: −0.070±0.82 (95% CI), t(17) = 0.18, p = 0.85, Cohen's d = 0.0026).

Experiment 3e showed that “far” faces were indeed rated as more Average (1.79±1.30, t(17) = 2.90, p<0.01, Cohen's d = 0.18). The averageness and trustworthiness ratings across all 36 faces (the 18 close and 18 far versions of each of the 18 individuals) were negatively correlated (r(34) = −0.36, p<0.05), resulting in residualized trustworthiness ratings (regressing out averageness) that still showed a significant effect of distance as before (2.30±1.47(95% CI), t(17) = 3.29, p<0.005, Cohen's d = 0.24). Similarly, competence ratings were slightly negatively correlated with averageness ratings (r(34) = −0.22, p = 1.9) and regressing out averageness did not significantly influence the effect of distance (2.14±1.88 (95% CI), t(17) = 2.40, p<0.03, Cohen's d = 0.21). Finally, attractiveness ratings showed the same pattern: negative correlation with averageness (r(34) = 0.51, p<−0.002) and regressing out averageness did not significantly influence the effect of distance (3.85±2.53 (95% CI), t(17) = 3.21, p<0.01, Cohen's d = 0.33).

A minority (16.4%) of participants in Experiment 3 indicated in the exit survey that they noticed a change in the face stimuli between trials. Excluding these participants from the analysis did not change any the results significantly.

### Discussion

Experiment 3 demonstrated that the influence of perspective distortion is robust even when administered over the internet, where display size and distance to the display are not controlled. Explicit manipulation of perceived distance to the face stimulus through image size or verbal instruction failed to show any effects, indicating possibly unique effects of perspective distortion as an implicit distance cue.

Perceptions of averageness were also influenced by perspective distortion, suggesting the possibility that these might in part mediate the effect on trustworthiness. However, across all of the 36 faces (close and far ones), averageness ratings were in fact anticorrelated with positively valenced trait ratings, with the result that regressing out the effect of averageness did not change the significance of the distance effect. However, it is important to note that there may be structural aspects of the face, such as its objective averageness, of which viewers are not explicitly aware, yet that could still influence social judgments. It will be important in future studies to experimentally manipulate structural averageness of faces independently of distance warping to definitively disentangle these effects.

Finally, as in Experiment 1, the participants in Experiment 3 incorrectly judged the camera distance of the stimuli. This result bears further investigation, but at minimum rules out the possibility that our results are mediated by accurate, explicit representations of interpersonal distance. Given how consistently perspective projection affects our results, however, we suggest that an implicit processing mechanism may be responsible for these effects on rapid social judgments.

## Experiment 4

Due to the constraints of generating the stimuli as well as time taken during the experiment, all of the above experiments relied on a relatively small set of 18 base faces (or synthetically warped versions thereof). In order to further verify the generality of our findings, we conducted Experiment 4, which replicated our results with a completely different set of face images that were also photographed under more naturalistic conditions. We collected 18 new photographs at close and far distances from a photographer, all taken outdoors in ambient daylight.

### Results

In Experiment 4, the far faces elicited higher ratings of attractiveness (2.51±1.04, t(17) = 2.49, p<0.03, Cohen's d = 0.19), competence (1.88±0.80, t(17) = 2.42, p<0.03, Cohen's d = 0.16), and trustworthiness (2.87±1.13, t(17) = 2.61, p<0.03, Cohen's d = 0.26) compared with those photographed at the closer distance.

### Discussion

Experiment 4 replicated the basic findings of the prior experiments with a new set of faces, supporting the hypothesis that the rapid evaluation of social traits such as attractiveness, trustworthiness, and competence are subject to the influence of perspective projection. The inclusion of additional faces in the experiment also allows us to aggregate the data over both stimulus sets. Combining the trustworthiness ratings from Experiment 1 with the trustworthiness ratings of Experiment 4 results in ratings for a larger dataset of 36 faces, which display an average Far-Close trustworthiness rating of 3.15±1.71 (95% CI), t(35) = 3.73, p<0.0001, Cohen's d = 0.24.

## General Discussion

We report a reliable novel effect, replicated across three stimulus sets and two different sets of base faces, several different experimental settings, and several subject samples: viewers prefer faces photographed from outside of personal space more than those photographed from within it. The effect was found in an economic trust game played with real money, in ratings gathered under laboratory conditions, and in ratings gathered over the internet. It was found for social judgments encompassing trustworthiness, competence and attractiveness. Geometric warping of the face alone (modeling perspective distortion due to distance) accounted for the effect while controlling for size, expression, resolution, highlights, focus, and explicit knowledge of camera distance.

Faces photographed at the far distance (135 cm) were also rated as more average. Given that all these ratings are intercorrelated to some extent, it is difficult to determine which of these judgments might possibly be mediating any of the others; for instance, it is plausible that the perceived averageness of the faces in part drives the differences in trustworthiness that we report. However, when controlling for averageness, the effect of distance on trustworthiness judgments in fact increased (Experiment 3), indicating that our distance manipulation does not influence trustworthiness judgments derivatively merely by altering perceived averageness. On the other hand, it remains possible that viewers were inaccurate in their judgments of averageness, an issue that future studies with objective measures and manipulations of averageness would be required to resolve.

It also remains unclear to what extent the effect we found is driven primarily by a particular social judgment. For instance, it is possible that there is a specific effect of distance on trustworthiness evaluations; but it is also possible that the effect operates on attractiveness judgments, and these secondarily influence other social judgments such as trustworthiness. Given the close intercorrelation between these judgments and the relatively small sample of faces in our studies, it was not possible to disentangle this. We consider it likely that perspective distortion influences several social judgments, and that it is not limited to trustworthiness alone.

It is likely that the cue of perspective distortion from distance usually operates implicitly, as it did in our experiment. Participants were incorrect when asked to judge camera distance, and post-experiment questioning showed that participants were unaware of any manipulation in facial appearance from trial to trial. The implicit nature of our distance cue is intriguing not only because it isolates psychological processes that could otherwise be contaminated by overt reasoning about distance, but also because the two explicit distance cues we examined (image size and verbal information) in fact did not produce effects on trustworthiness judgments.

There is a documented effect of facial masculinity proportions (the face width-to-height ratio) on perceived untrustworthiness (5). However, this is unlikely to account for our finding as the facial width-to-height ratio is actually smaller in our “close” than “far” faces (paired t-test, t(17) = 11.16, p<0.001); if width-to-height ratio were the predominant effect, it would lead to an effect in the direction opposite from what we observed. Face warping from projection distance thus appears to be an independent signal used for social judgments.

The importance of the present findings extends beyond our discovery of a novel social cue from faces. Perspective distortion is perhaps the first implicit cue to interpersonal distance, opening the door for further studies on the underlying psychological processes as well as the brain structures involved in the automatic evaluation of personal space. Attractive aspects of perspective distortion, as a cue to social judgments, are that it has a natural parameterization and that it may be studied in isolation from other cues.

Future applications could be to predict, and to manipulate, viewers' feelings about other people from quantification of the perspective distortion of photographs on the internet, in magazines, and in personal identification documents (8). An important limitation of the findings thus far concerns their generality: the literature documents many variables that interact with personal space. No doubt, there will be effects of gender [31] and familiarity (17), of culture [17], [32], of the facial expression and of the context in which the face is seen [33], [34], all of which are likely to interact with the perspective factor we isolated here.

## Experiment 1 Methods

### Subjects

(see Table 1 for an overview). Healthy adult participants were recruited from the local community through posted flyers and Internet ads. Experiment 1a: N = 23, mean age = 33.26±2.92 (SEM), (17 female, 6 male; 7 White, 6 Asian, 4 Hispanic, 1 African-American, 5 Other). Experiment 1b: N = 45, mean age = 25.91±1.18 (SEM), (35 female, 10 male; 34 White, 6 Asian, 3 Hispanic, 2 African-American), Experiment 1c: N = 37 (23 female, 14 male;). Participants in Experiment 1a were non-overlapping with those in Experiments 1b,c whereas all of those in 1c had first participated in 1b.

### Ethics Statement

All participants gave written informed consent in compliance with Caltech's Institutional Review Board, which specifically approved the study (IRB number RA-127). Data were analyzed anonymously and in aggregate, after renormalization to a common scale. The participant shown in Figure 1 has given written informed consent, as outlined in the PLoS consent form, for publication of their photograph.

#### Stimuli

**Figure 4:**
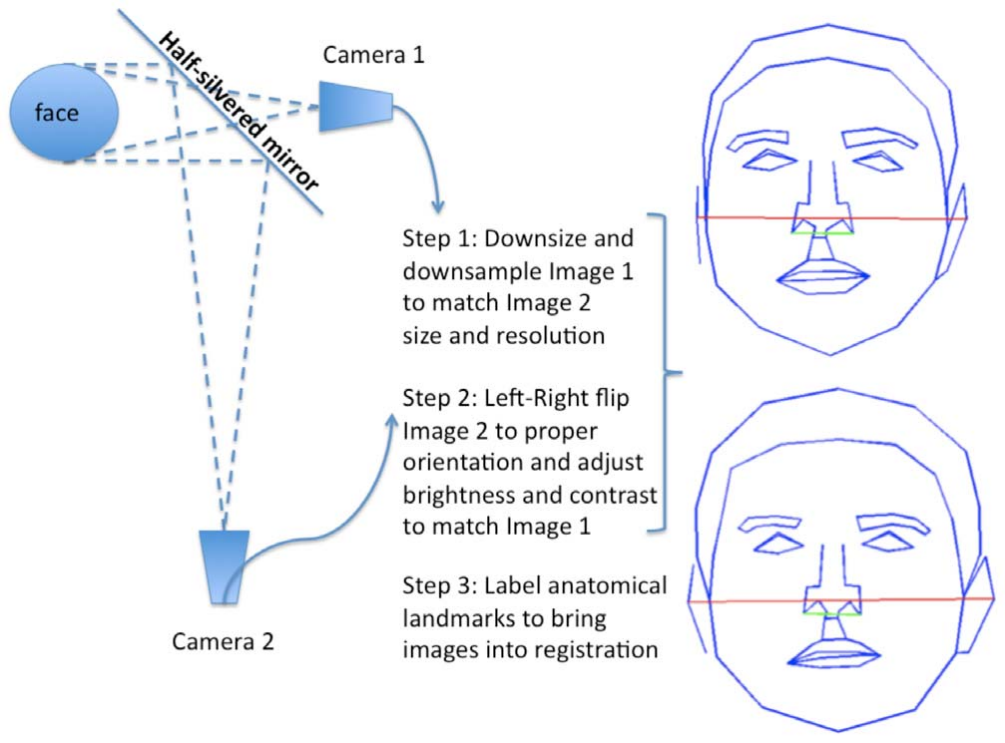
Creation of stimuli. The schematic illustrates how the two photographs of a face were taken at two distances simultaneously, and summarizes the post-processing steps to equate the resultant images and generate the stimuli.

Participants viewed frontal grayscale photographs of the faces of 18 unfamiliar White males, Age = 33±12, displaying direct gaze and a neutral expression. For each face, two photographs were taken simultaneously from distances of 45 cm and 135 cm using a half-silvered mirror, which ensured that the facial expression would be identical (Figure 4). The distances were chosen to be within and outside of personal space, respectively (10). Camera alignment was confirmed with a digital laser meter; lens distortion of checkerboard test images was negligible. The far image, captured after reflection on the mirror, was left- right flipped to restore the original orientation. The close image was downsampled and resized to match the resolution and dimensions of the far image. Both images were converted to grayscale and set to the same luminance and contrast. Size was equated by equating interocular separation. Each image was rotated so that the eyes were perfectly aligned horizontally and placed at the vertical center of the screen. All stimuli were shown on an LCD monitor, presented for 5 s (Experiment 1a) or 2 s (Experiments 1b,c) at 11.4 degrees visual angle in a normally lit room.

### Procedure

Participants were tested individually in the lab and viewed images on a computer monitor using a fixed-distance chin rest. In Experiment 1a, participants played an economic trust game [35], a tool used in behavioral economics [36] that reliably measures trust [37]. Participants were given a $100 endowment of which they could invest any portion in a trustee, whose photograph was shown as the stimulus image. The amount invested was tripled and the portion returned to the participant was selected from previously recorded actual choices of the trustees whose faces we had photographed. Participants knew this and were told that one randomly selected trial would be implemented at the very end of the experiment, and would contribute to their actual cash payout. The incentive to participants was thus to genuinely try to estimate the trustworthiness of the trustees whose faces they were shown, in order to maximize their real earnings.

Participants first played a round against a computer to ensure they understood the instructions of the trust game. After that, they were introduced to the real experiment as follows, “…now you will see images of people's faces. You may have a first impression, an immediate gut reaction about whether or not you would like to invest with them. That is what we want you to pay attention to when you make your decision. One trial will be selected at random to determine a real payout. … We've asked the people who appear in these photos how much they would actually keep and return for each possible investment amount you can make, and we will use these responses in addition to your investment to determine how much you will actually make in this game. At the end of the experiment, we will give you a percentage of this amount. Treat every trial as if real money were at stake.”

In Experiment 1b, participants rated the faces on Trustworthiness, Competence, and Attractiveness on a 7-point scale (blocked by trait), and in 1c on Age, Weight, and Distance to the camera (always rated last to avoid the possibility that explicit attention to camera distance might impact other ratings). We asked participants to use the entire rating scale, and not to overthink the ratings but rather go with their gut feeling if they were unsure. Participants were also told ahead of time that all faces would be of Caucasian males, so that their social judgments could be relative to this group of people from the outset. Finally, participants were explicitly told that some of the stimuli would be repeated, and that we were interested in how their responses might change over time; they should therefore feel free to vary their responses and not make any attempt to memorize what response they gave to the prior occurrence of a given face.

We defined “attractiveness” as relating to physical attractiveness, with a rating of 1 denoting a face that looks physically very unattractive and a 7 one that looks physically very attractive. We defined “competence” as relating to the person's likely ability at their job, with 1 meaning they are incompetent at their job, and 7 meaning that they are very competent at doing their job. We defined “trustworthiness” as relating to moral character and in particular how much you would trust them with a large sum of money to hold safe for you.

Participants viewed all 18 faces twice in each distance condition. Faces were presented in randomized order, but distance pairs were counterbalanced across quarters of the experiment such that half the faces were viewed first in the close condition followed by the far condition. Dollar investment amounts in studies 1 and 2 and raw ratings from all three studies were normalized to a 1–100 scale for subsequent analyses, based on each participant's individual range across all faces.

## Experiment 2 Methods

### Subjects

N = 27, mean age = 23.93±1.09 (SEM), (17 female, 10 male), (15 White, 7 Asian, 1 Hispanic, 1 African-American, 3 Other) recruited from the local community in the same manner as Experiment 1.

### Stimuli and Procedure

Photographs of faces from Experiment 1 taken at 135 cm were warped to the proportions of those taken at 45 cm (Figure 2b). Warping was accomplished by manually labeling 115 anatomical facial locations (including eyes, nose, mouth, ears, and outline) and interpolating using Delaunay triangulation, a standard technique for digital morphing. Thus the location coordinates of major anatomical features are exactly the same for the close faces in Experiment 1 and Experiment 2, but the luminance values are slightly different. The average 2D correlation between the pixel values of a close face in Experiment 1 and its corresponding synthetic warp in Experiment 2 is quite high (r = 0.95±0.004 (SEM)), indicating that the role of these subtle luminance differences may in fact be negligible. Participants performed the same economic trust game as in Experiment 1a.

## Experiment 3 Methods

### Subjects

Participants were recruited only from the United States and tested over the internet via Amazon's Mechanical Turk, permitting larger sample sizes (Experiment 3a, N = 268, 148 female; Experiment 3b, N = 70, 27 female; Experiment 3c, N = 60, 27 female; Experiment 3d, N = 253, 143 female; Experiment 3e, N = 134, 68 female).

### Stimuli

Experiment 3a, 3d, 3e, and 3f all used identical stimuli as Experiment 1.

Experiment 3b used only the “far” stimuli from Experiment 1, but accompanied by a verbal cue to distance before presentation indicating that the person was “standing 1.5 feet in front of you” or “standing 4.5 feet in front of you.” Experiment 3c used only the “far” stimuli from Experiment 1, but adjusted the size of the image to take up the entire screen or just half of it.

### Procedure

Experiments 3a,b,c obtained the same ratings as in Experiment 1b: trustworthiness, competence, attractiveness. Whereas Experiment 3a showed the identical stimuli as in Experiment 1b (strictly replicating that lab-based experiment), Experiment 3b used only the “far” faces accompanied by a verbal cue to indicate that the person was standing either near or far, and Experiment 3c showed the “far” faces at 2 different screen sizes. Experiments were administered in fixed order, 3a,b,c.

Experiment 3d obtained the same ratings as in Experiment 1c: age, weight, and distance to the camera; Experiment 3e obtained ratings of how average, and how animal-like the faces appeared. These Experiments were also administered in fixed order, 3a,d,e. See Table 1 for more information about all the experiments.

## Experiment 4 Methods

### Subjects

N = 31, mean age = 31.8±1.55 (SEM), (17 female, 12 male), (18 White, 9 Asian, 4 Hispanic,) recruited from the local community in the same manner as Experiment 1.

### Stimuli and Procedure

Photographs of 22 new faces (all white males) were acquired using conventional methods, sequentially photographing close and far (counterbalanced across subjects so that half the faces were photographed close first and the other half were photographed far first). All photographs were taken outside in ambient daylight by a male photographer with a digital SLR camera. The distances in Experiment 4 were slightly different than in the other experiments as well: the close faces were photographed from 54 cm and the far faces were photographed from 120 cm. The digital images were equated for mean contrast, luminance and interocular separation as before, and presented for 2s at 11.4 degrees visual angle. Participants rated Attractiveness, Competence, and Trustworthiness in a blocked design similar to that described for Experiment 1b. Due to the fact that the faces were not photographed simultaneously, some of the stimuli displayed subtly different facial expressions. We asked two independent viewers to rate the facial expressions of the photographs, and excluded 4 pairs that were rated as discordant in expression by both raters, resulting in a final stimulus set of 18 faces.
